# Machine Learning for Multimedia Communications

**DOI:** 10.3390/s22030819

**Published:** 2022-01-21

**Authors:** Nikolaos Thomos, Thomas Maugey, Laura Toni

**Affiliations:** 1 School of Computer Science and Electronic Engineering, University of Essex, Colchester CO4 3SQ, UK; 2 Inria, 35042 Rennes, France; thomas.maugey@inria.fr; 3 Department of Electrical & Electrical Engineering, University College London (UCL), London WC1E 6AE, UK; l.toni@ucl.ac.uk

**Keywords:** multimedia communications, machine learning, video coding, image coding, error concealment, video streaming, QoE assessment, content consumption, channel coding, caching

## Abstract

Machine learning is revolutionizing the way multimedia information is processed and transmitted to users. After intensive and powerful training, some impressive efficiency/accuracy improvements have been made all over the transmission pipeline. For example, the high model capacity of the learning-based architectures enables us to accurately model the image and video behavior such that tremendous compression gains can be achieved. Similarly, error concealment, streaming strategy or even user perception modeling have widely benefited from the recent learning-oriented developments. However, learning-based algorithms often imply drastic changes to the way data are represented or consumed, meaning that the overall pipeline can be affected even though a subpart of it is optimized. In this paper, we review the recent major advances that have been proposed all across the transmission chain, and we discuss their potential impact and the research challenges that they raise.

## 1. Introduction

During the past few years, we have witnessed an unprecedented change in the way multimedia data are generated and consumed as well as the wide adaptation of image/video in an increasing number of driving applications. For example, Augmented Reality/Virtual Reality/eXtended Reality (AR/VR/XR) is now widely used in education, entertainment, military training, and so forth, although this was considered a utopia only a few years ago. AR/VR/XR systems have transformed the way we interact with the data and will soon become the main means of communication. Image and video data in various formats is an essential component of numerous future use cases. An important example is intelligent transportation systems (ITS), where visual sensors are installed in vehicles to improve safety through autonomous driving. Another example is visual communication systems that are commonly deployed in smart cities mainly for surveillance, improving the quality of life, and environmental monitoring. The above use cases face unique challenges as they involve not only the communication of huge amounts of data, for example, an intelligent vehicle may require the communication of 750 MB of data per second [1], with the vast majority of them being visual data, but they also have ultra-low latency requirements. Further, most of these visual data are not expected to be watched, but will be processed by a machine, necessitating the consideration of goal-oriented coding and communication.

These undergoing transformative changes have been the driving force of research in both multimedia coding and multimedia communication. This has led to new video coding standards for encoding visual data for humans (HEVC, VVC) or machines (MPEG activity on Video Coding for Machines), novel multimedia formats like point clouds, and support of higher resolutions by the latest displays (digital theatre). Various quality of experience (QoE) metrics (also known as quality factors) have been proposed considering not only the quality of the delivered videos but also parameters such as frame skipping, stalling, and so forth, and the fact that consumers may request the same data but consume them differently. The realization that consumers of visual data can be machines has initiated discussion regarding the definition of task-oriented quality metrics. Since end-users’ demands for multimedia content are diverse, and users interact with the content differently, multimedia communication systems consider users’ behavior and employ cloud/edge/fog/device caching and computation facilities to allow content reuse. The extremely tight latency requirements of the latest multimedia application render impossible having retransmission mechanisms (ARQ) or access to accurate channel estimations. The joint consideration of source and coding has been revisited to design systems that meet the expectations of future multimedia coding and communication systems. These landscaping changes result in complex decision-making problems that conventional optimization methods cannot solve. Further, they are closely related to resource provisioning and the prediction of future trends. Therefore, they naturally call for defining machine learning-based systems that can address the challenges future multimedia coding and communication systems face.

Nowadays, machine learning (ML) is commonly used in multimedia communication systems. Machine learning-based coding for images [2] and video [3,4] is explored by standardization communities as a potential solution for achieving more efficient compression or compression oriented to tasks performed by machines. Apart from compressing visual sources, machine learning is used to predict the data quality at the receiver as well as users’ consumption patterns. Further, machine learning-based prediction algorithms are developed for resource provisioning, data prefetching, caching, and adaptation of channel protection. Machine learning is also used to further boost the performance of the image/video codecs avoiding the excess computational cost of the latest coding standards. This survey focuses not on providing a comprehensive overview of the literature for each part of the multimedia encoding and delivery ecosystem but on introducing the main challenges, emphasizing the latest advances in each area, and giving our perspective for building more efficient intelligent multimedia coding and delivery systems. For surveys focusing on particular parts of this ecosystem, interested readers are referred to [5,6,7,8] for compression/decompression, ref. [9] for interactivity, refs. [10,11] for modeling users’ behavior, and refs. [12,13,14] for video caching, and so forth. These surveys concern works employing machine learning algorithms to address the challenges of only a part of the ecosystem.

Although machine learning-based multimedia communication and coding systems achieve significant gains in comparison to conventional systems, the main sources of suboptimality are that: (i) these research areas have been studied and developed in a fragmented way; (ii) coding and communication frameworks remain human-centric while an increasing number of applications are machine-centric; (iii) interactivity is not fully taken into account when designing an end-to-end multimedia communication system; (iv) machine learning-based image/video coding systems still rely on entropy coding, which compromises error-resilient properties and complicates the transport protocols; (v) the multimedia delivery systems have been optimized considering the structure of the bitstream generated by conventional image/video coders and, hence, perform suboptimally when used to transport bitstreams generated by machine learning-based image/video coding systems; and, (vi) the latest use cases, for example, ITS, AR/VR/XR, and so forth, have ultra-low latency requirements that cannot be met by optimizing a part of the ecosystem separately. In this survey, we advocate for the need for machine learning-enabled end-to-end multimedia coding and communication systems that consider both human and machine users that actively interact with the content.

In the following, in Section 2 we present the typical video and image coding and communication pipeline and discuss why machine learning is needed for multimedia communications to achieve superior performance. Then, in Section 3 we explain the main parts of a multimedia coding and communication system, presenting the recent attempts to use machine learning for building more efficient systems. In Section 4, we discuss the main challenges and outline our vision towards machine learning-enabled end-to-end multimedia coding and communication systems. Finally, conclusions are drawn and future directions are outlined in Section 5.

## 2. Multimedia Transmission Pipeline

**Figure 1:**
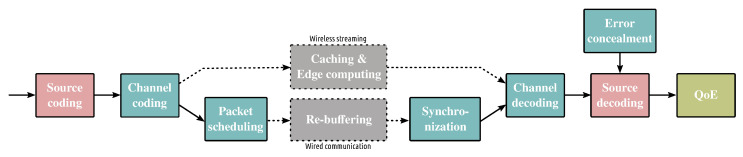
Multimedia transmission pipeline.

A high-level representation of a multimedia coding and communication system is depicted in Figure 1. The illustrated blocks are shared by the main types of multimedia data, that is, images [15,16] and video [17,18,19], which are essential components of other types of pictorial data. Source coding aims at removing the spatial redundancy for images and spatio-temporal redundancy for videos. This is achieved by transforming the pictorial information into a sparser domain using DCT or DWT transform, for example. Sparsity permits to greatly compress the information. The transform can be either applied to the entire image or blocks. The visual information is quantized prior to compress it. Through quantization, the most important information for the human visual system is maintained while the rest is removed. Entropy coding (arithmetic coding, Huffman coding) [20] is used to compress the generated multimedia bitstreams by allocating shorter representation to more common bitstream patterns and longer to less frequent. For video coding, additional tools are used to exploit the temporal correlation as video streams are a sequence of correlated images. Residual coding is exploited for this reason. In particular, before applying residual coding, motion estimation and motion compensation are used to generate frames that are subtracted from the original frames to generate the error frames that are eventually compressed to minimize the amount of encoded data. Depending on the source data, other tools may be applied to compress the data efficiently.

After source coding, the multimedia data can be either transmitted over wireless or wired connections. For wireless transmission, the data are protected by means of channel coding such as Turbo codes [21], LDPC codes [22], Polar codes [23], Reed Solomon codes [24], to name a few. The employed channel code depends on the anticipated channel conditions and the type of expected errors, that is, bit errors or erasures. Channel codes add redundancy to the transmitted bitstream so that they can cope with the errors and erasures. The information can be either protected equally or unequally. The latter is preferable when some parts of bitstream have higher importance than the rest, as in image and video cases. The last step before transmitting the multimedia bitstreams is modulation, which aims at improving the throughput and at the same time making the bitstream more resilient to errors and erasures. As wired communication do not experience bit errors, but packet losses, the most popular communication protocols for multimedia delivery (HTTP streaming [25,26], WebRTC [27], etc.) have native mechanisms (TCP) to deal with them by requesting erased or delayed packets through retransmission mechanisms avoiding the use of channel codes as we will explain later. However, in the absence of retransmission mechanisms, as is the case in peer-to-peer networking [28], erasure correcting codes such as Reed Solomon codes, Raptor codes [29], and so forth, are still used to recover erased packets.

**Figure 2:**
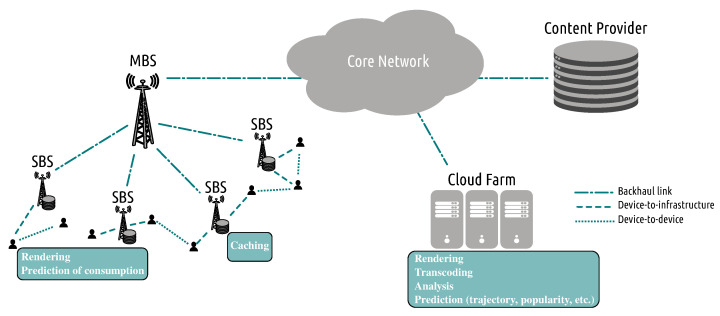
Communication ecosystem.

The delivery of the multimedia bitstreams can be facilitated by edge caching [30] and cloud/edge/fog facilities [31,32,33]. The former is used to exploit possible correlations in the users’ demands. This is the case as a small number of videos accounts for most of the network traffic. Through edge caching, as illustrated in Figure 2, the most popular information is placed close to the end-users, from where the users can acquire it without using the entire network infrastructure. Cloud/edge/fog infrastructure is mainly used to process the information, for example, to predict the most popular data or to process the data, for example, video transcoding [34] or convert the media formats used by different HTTP streaming systems [35] to minimize the amount of information cached and thus better use caching resources. For the case of wired transmission (i.e., HTTP streaming, WebRTC, etc.), packet scheduling decisions are made to decide on how to prioritize the transmitted packets so that the most important information is always available to the end-users.

At the receiver end, the reverse procedure is followed to recover the transmitted multimedia data. Hence, in case of wireless transmission, the received information is first demodulated and then is channel decoded. To cope with remaining errors after channel coding, the video decoders activate error concealment, which aims at masking the bit errors and erasures by exploiting temporal and spatial redundancy. Another critical video coding tool that is activated to improve the quality of the decoded videos is loop filtering that aims at combating blocking artifacts occurring at the borders of the coding blocks. As the packets may arrive at the receiver out of order due to erasures, the video codecs resynchronize the packets respecting the playback timestamps and may request retransmission of packets that did not reach their destination. Packet rebuffering depends on the affordable time to display the information. The quality of the transmitted data is measured using QoE metrics such as PSNR, SSIM, MOS, Video Multi-method Assessment Fusion (VMAF) [36,37] for the video case, etc. Apart from these metrics for videos, other factors are considered, such as video stalling, frame skipping, delay, among others.

Multimedia communication efficiency has constantly improved over the last decades, due to intensive research efforts and new hardware designs. However, several key aspects of the multimedia processing pipeline still rely on hypotheses or models that are too simple. As an example, the aforementioned DCT used in most of image/video coders is optimal when the pixels of the image follow some given distributions (e.g., Gaussian Markov Random process [38]) that is almost never verified in practice. As a second illustrative example, in spite of numerous studies, QoE metrics still do not manage to properly capture the human visual system and the subjectiveness of the multimedia experience. Such observations can be made for every part of the transmission pipeline. This is the reason why research on multimedia communication has recently been oriented towards the power of machine learning tools. Indeed, learning-based solutions have generated an unprecedented interest in many fields of research (computer vision, image and data processing, etc.) as they enable us to capture complex non-linear models in deep neural network architectures [39]. In the next section, we review how learning-based approaches have been investigated in multimedia transmission.

## 3. Learning-Based Transmission

### 3.1. Compression/Decompression

**Figure 3:**
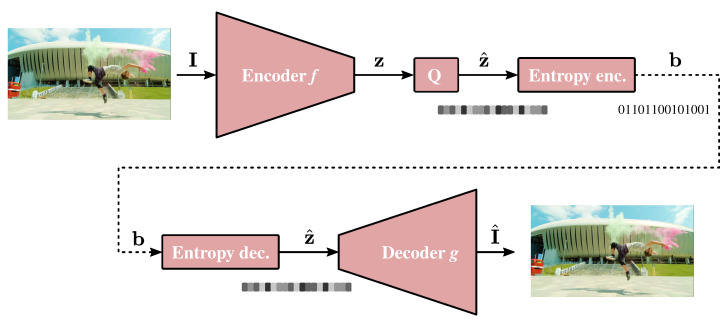
Usual architecture of end-to-end learning-based compression algorithms. The encoding and decoding functions *f* and *g* enables us to project the multimedia signal into a latent space of reduced dimension. The quantization *Q* and the entropy coding aims at describing the latent vector into a compact binary stream.

The recent arrival of learning-based solutions in the field of image compression has kept its promises in terms of performance. It is nowadays proven that end-to-end neural networks are able to beat the most performing image compression standards such as JPEG [15], JPEG 2000 [40], BPG [41], AV1 [42] or VTM [43]. The field has been very active, and more and more performing architectures, on top of extensive surveys, are regularly proposed [5,6,7,8]. Most of the existing architectures share the same goal and principles that are depicted in Figure 3. They completely get rid of the existing coding tools and perform the compression thanks to dimensionality reduction *f*, based on a deep neural network architecture. The obtained latent vector z is then quantized to $\hat{z}$ and coded using an entropy coder, also usually relying on deep learning architectures. After entropy decoding of the bitstream, the latent vector is finally decoded, again, using a multi-layer neural network *g*. In such a system, the goal is to obtain a binary vector b as small as possible, and at the same time, a minimum decoding error, that is, small distance $d(I,\hat{I})$, where I and $\hat{I}$ stand for the original multimedia content and the reconstructed content, respectively. Authors in [44] propose to practically derive the rate-distortion bounds of such non-linear transform approaches, while the authors of [45] have built an open-source library to assess the most meaningful architectures. Even more gigantic compression gains can be reached by changing the definition of the reconstruction error. As shown in various studies [46,47,48], the classical mean squared error does not reflect the perceptual quality of a compressed image properly, especially at low bit-rates. For these reasons, the most recent architectures achieve even higher compression rates by changing the ultimate compression goal [49,50].

For video compression, the benefits of learning-based solutions have been less immediate, mostly because temporal motion is still not accurately modeled by neural network architectures. Naturally, deep neural networks (DNNs) can be used to assist the complex encoding/decoding operations performed by the standard video coders, or to post-process the decoded video [51,52]. Entirely replacing the video coder with an end-to-end system is less straightforward. The existing learning-based approaches [53,54,55,56] still use handcrafted tools such as motion estimation or keypoint detection. Some very recent works have however tried to avoid the use of the classical predictive structure involved in a classical video coder [57]. A detailed review is presented in [58].

Learning-based approaches have also been explored to compress other types of multimedia contents, such as 360-degree images [59], 3D point clouds or meshes [60,61,62]. The main issues are to deal with irregular topology, thus requiring to redefine the basic tools composing a neural network architecture, for example, convolution, sampling. They can rely on recent advances in the so-called geometric deep learning field [63], which mostly study how to apply deep learning on manifolds or graphs.

To summarize, learning-based approaches reach groundbreaking compression gains and further impressive coding efficiency can be expected in the coming years. However, deep learning architectures remain heavy and sometimes uncontrollable. Some recent works such as [64] have focused on a better understanding of deep neural networks and, in particular, on the decrease of complexity and better interpretability.

### 3.2. Error Resilient Communication

#### 3.2.1. Channel Coding/Decoding

Commonly, the communication of the video data happens over error-prone channels such as Binary Symmetric Channels (BSC), Binary Erasure Channels (BCE), and Additive White Gaussian Noise (AWGN) channels, which may lead to corrupted frames, stalled frames, error propagation, etc. and, eventually, degradation of users’ quality of experience. In order to avoid such QoE degradation, channel coding is used to protect the communicated video data. There exist many efficient channel codes like Low Density Parity Check (LDPC) codes [22], Turbo codes [21], and Polar codes [23], Reed Solomon codes [24], and so forth. These codes achieve a performance very close to the Shannon limit, but only for large codeblock lengths, for transmission over BSC, BCE and AWGN channels, and when relatively long delays are affordable [65]. Due to their efficiency, LDPC codes and Polar codes have been adopted by the 5G New Radio (5G-NR) standard and are used for protecting the data and control channels, respectively. However, the emergence of new communication paradigms such as Ultra-Reliable Low-Latency Communication (URLLC) and Machine Type Communication (MTC) challenge the existing channel codes. In these paradigms, the packet lengths are small to medium, and the affordable delays are very tight. Similar challenges are also faced by VR and AR systems, as typically, these systems have extremely tight delivery delays and, thus, the employed packet lengths cannot be large.

The challenges above triggered significant research in redesigning channel encoding/decoding processes so that the decoding performance is maintained high for short and medium codeblock lengths, and both the decoding complexity and the decoding delay are greatly reduced. To this aim, machine learning has been proposed for channel encoding [66,67,68], channel decoding [69,70,71,72,73,74,75,76,77,78,79], and building end-to-end communication systems where channel encoding and decoding are jointly considered [80]. Different machine learning methods have been examined, for example, neural networks are used in [69,70,71,72,73,74,75,76], while designs based on reinforcement learning methods are presented in [77,78,79].

The design of LDPC codes is usually done using EXIT charts or density evolution, however for short codeblock lengths, the assumptions made by these methods do not hold anymore. This fueled the research on designing well-performing channel codes for the non-asymptotic case. In [66], a concept inspired by actor-critic reinforcement learning methods is presented. The proposed framework is based on a code constructor, who builds a channel code (parity check matrix), and a code evaluator, who evaluates the efficiency of the designed code. The code constructor keeps improving the code structure until a performance metric converges. Three different methods were examined for the design of the codes, namely Q-learning, policy gradient advantage actor-critic, and genetic algorithms. This framework is generic and applicable for linear block codes and Polar codes. The use of genetic algorithms for designing efficient channel codes has also been examined in [67] where the target is short codeblock lengths. These codes outperform 5G-NR LDPC codes for that range of codeblocks. The designed codes can be tuned to achieve lower decoding complexity and latency with only a small degradation in the decoding performance. Tunability of the code design is also a subject of the LDPC design in [68] where the density evolution is mapped to a recurrent neural network (RNN).

Machine learning-based channel decoding is proposed to reduce the decoding complexity and improve the decoding performance of High-Density Parity Check (HDPC) codes for medium to short codeblock lengths. Replacing the belief propagation decoder used by HPDC (and LDPC) decoder with a decoder based on neural networks, known as neural belief propagation, has been first presented in [71,73,74]. Initially, feedforward networks were considered [73,74], while later these are replaced by recurrent neural networks [71] for improved decoding performance. The underlying idea is to map each decoding iteration of the belief propagation decoder (which corresponds to the Tanner graph) to two hidden layers of a neural network and then assign trainable weights to the neural network. Then, training is done by means of the stochastic gradient descent algorithm. The decoder achieves improved decoding performance and reduced complexity. To further reduce the complexity of the decoder, the design is applied to the min-sum decoder and a neural normalized min-sum decoder was presented. Lower complexity is achieved because the magnitudes of the messages exchanged in the min-sum decoder are smaller. Parameters tying and relaxation are also proposed to boost the decoding performance further. More recently, active learning is proposed in [69] to improve the decoding performance through selecting the most appropriate samples for training. Linear approximation of the messages exchanged by the neural min-sum decoder is studied in [70] to reduce the size of the employed neural network and, hence, the number of trainable parameters. Similarly, the reduction of the number of trainable parameters for the neural min-sum decoder is studied in [72]. It is proposed to exploit the lifting structure of the protograph LDPC codes, which are the base codes of 5G-NR LDPC codes. Through parameters sharing, the number of parameters that should be learned are greatly reduced. In addition, the parameters are learned iteration by iteration, avoiding problems with vanishing gradients. Pruning the neural belief propagation decoder is proposed in [75] where weights show how much each check node (in the Tanner graph representation) affects the decoding performance. The message exchange schedule of the belief propagation decoder is modeled as a Markov Decision Process (MDP), and reinforcement learning is to find the optimal scheduling [77,78]. Fitted Q-learning has been used to map the low complexity bit-flipping decoding algorithm to an MDP so that the optimal bit-flipping strategy is determined [79]. As shown in [76], channel decoding can be seen as a classification problem, and a deep neural network can be used to perform one-shot decoding. It is shown that the system can generalize to unseen codewords for structured codes, while for random codes, this cannot be done. This scheme can be used only for small codewords, as the complexity of training grows exponentially with the number of information bits. One appealing characteristic of all machine learning-based channel decoding methods is that training can be done only using the zero codeword and adding random noise to it. This is due to the fact that the considered channels are symmetric and the weights of the belief propagation decoder are non-negative.

The joint channel encoding and decoding design is proposed in [80] to improve the resilience to channel mismatch conditions while maintaining latency similar to neural belief propagation. Specifically, the aim is at reducing the structural latency, that is, the time required from the reception of a codeword to the start of the decoding. The joint design is based on recursive neural networks. The result shows that the proposed design outperforms state-of-the-art tail-bitting convolutional codes and is robust to channel mismatch, but the superior performance cannot be maintained for large codeblocks.

#### 3.2.2. Error Concealment

Error concealment is a key component of video communication systems, as the communication is done over error-prone channels that introduce errors to the received bitstream. It aims at localizing the effect of an error and conceal it by exploiting temporal and spatial redundancy. For more efficient error concealment, tools such as motion copy, motion vector extrapolation, motion estimation at the decoder, exploitation of side information at the decoder, insertion of redundant video frames and redundant slices, intra-macroblock updates, and others can be used [81]. Some of the error concealment tools deteriorate the video coding performance, such as the insertion of redundant video frames or intra-macroblock updates, while others only require additional processing power at the decoder like motion vector extrapolation, motion estimation at the decoder, among others. More recent video coding standards such as HEVC and VVC have very limited error concealment support compared to predecessors, for example, H264/AVC and, hence, they generate video streams sensitive to errors. A way more recent compression standards achieve superior compression performance is by exploiting more the temporal dependencies. Hence, whenever a loss happens, there is a greater degradation in the video quality. The same conclusions apply to machine learning-based video coding methods as those cited in Section 3.1, where parts of the video compression pipeline are replaced by machine learning-enabled ones, as well as those where the entire pipeline has been replaced by a machine learning-based end-to-end video codec. The latter schemes do not have any error-resilient support and are very sensitive to errors, as they employ entropy coders for improved compression performance.

There is very limited literature on machine learning-based error concealment tools. Long short-term memory (LSTM) based optical flow prediction is proposed in [82]. This is a post-processing tool that is transparent to the underlying video codec. To control the complexity, it is proposed to perform only forward prediction, to limit the number of LSTM layers and to use only a portion of the optical flow vectors for prediction. The model is non-blind and requires knowledge of the location of the packet loss and the macroblocks above and below. The use of temporal capsule networks to encode video-related attributes is examined in [83]. It operates on three co-located “patches”, which are 24×24 pixel regions extracted from a video sequence. Patch-level motion-compensated reconstructions are performed based on the estimated motion, which are used for error concealment. Improving the error concealment support of HEVC is studied in [84]. To this aim, generative adversarial network (GAN) and convolution neural network (CNN) are employed. This method uses a completion network that tries to fool the local and global critics. For reduced complexity, it is proposed to focus on the area around the lost pixels (area affected by packet loss), which is done using a mask attention convolution layer that performs partial convolution. This layer uses pixels from error-free received pixels from the surrounding area in the previous frames.

More efficient concealment is achieved when advanced error concealment tools such as the ones we discussed previously are combined with optimized partitioning to coding units (CUs). By partitioning the CUs into smaller blocks that are encoded either in intra or inter mode, more resilient to errors video streams are generated as errors can be localized. Most of the existing coding machine learning-based CU partitioning methods [85,86] consider only intra mode, which introduces a coding performance penalty. These methods target to reduce the complexity introduced due to the partitioning, as this is one of the computational processing intensive components of HEVC and VVC coders. Improved coding performance can be achieved by allowing the CUs to be coded in inter mode. When this happens, the motion vectors of the adjacent prediction units and reference frames are used to determine the candidate motion vectors. This strategy helps to confine the error propagation to smaller areas. This is considered in [87], where a deep learning framework is presented for deciding the optimal intra and inter CU partition. The prediction is based on deep CNN based on multi-scale information fusions for better error resilience. A two-stream spatio-temporal multi-scale information fusion network is designed for inter coding to combat error propagation. This method tradeoffs complexity reduction with only a small rate-distortion loss.

### 3.3. Video Streaming

Streaming strategies have been challenged by the heterogeneity and interactivity of users consuming media content. Fetching high-quality media content to any final users, despite their displaying device (Ultra quality TV, tablet or smartphone), available connectivity (broadband, 4G, etc.) and interactivity level (multimedia, virtual reality, mixed reality) requires large amounts of data to be sent in a real-time communication scenario, pushing connectivity boundaries. This requires multimedia systems to operate at scale, in a personalized manner, remaining bandwidth-tolerant whilst meeting quality and latency criteria. However, inferring the network constraints, user requirements and interactivity (to properly adapt the streaming strategy) is not possible without proper machine learning tools. In the following, we provide an overview of the main machine learning-based video streaming systems used both in wireless and wired streaming and clearly show the advances that machine learning has unlocked within this content.

#### 3.3.1. DASH-ABR

**Figure 4:**
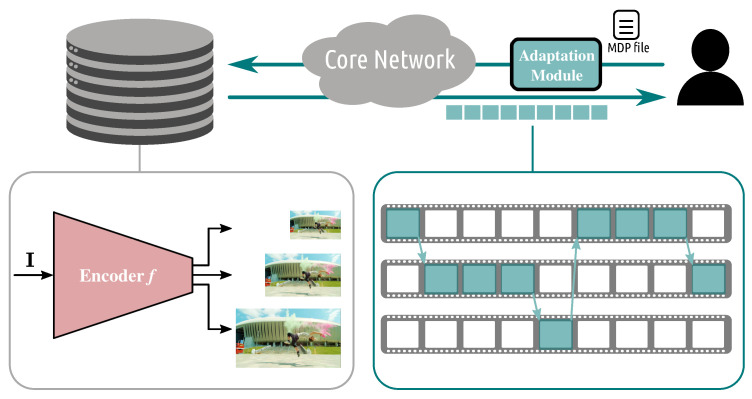
Adaptive video streaming over HTTP. After encoding the video into multiple representations (e.g., resolutions, qualities, etc.), it is stored in a server from where it can be delivered to the users. Before watching the video, users first obtain the MDP file, which contains information where the video is stored (typically, the video is split into chunks of 2–5 s), it acquires the video. The representation of the video displayed to the users is decided by the users and depends on the encountered channel conditions and other quality factors. The adaptation logic can be either based on control theory approaches or machine learning. The latter permits the consideration of multiple quality factors and forecasting future changes in the network conditions.

*Adaptive streaming over HTTP* addresses the increasing and heterogeneous demand for multimedia content over the Internet by offering several encoded versions for each video sequence. Each version (or *representation*) is characterized by a resolution and a bit rate. Each representation is decomposed into temporal chunks (usually 2 s long) and then is stored at a server. This means that at the server side, each chunk of a given video is available into multiple encoded versions. The main idea behind this strategy is that different users will have different quality and bandwidth requirements and will download different representations, which is decided by an adaptive bitrate (ABR) algorithm. The selection of the optimal representation for a given user happens at the client side, where the intelligence stands as a form of *adaptation logic* algorithm. Specifically, a client watching a media content will receive a media presentation description (MPD) file from the server, which contains information about all the available representations for each content. Given this information, the client requests the best set of representations for the current chunk based on the available bandwidth, playback buffer, and so forth. The downloaded representations are stored at the client side buffer, from where the chucks are extracted for decoding and displaying. Requesting the best representations means finding the optimal tradeoff between asking for high-quality content and asking for not-too-high bitrate. While a high-bitrate increases the quality of the decoded media content, it also requires large bandwidth to sustain the download. Should this not be available, the user might encounter into large downloading delay, risking emptying the buffer and experience *rebuffing* (or stalling) events, with a negative impact on the quality of experience. The main components of an adaptive streaming over HTTP system are depicted in Figure 4. In the following, we describe key machine learning-based ABR solutions. We first focus on works aimed at inferring the network and system conditions, with an initial focus on frameworks aimed at explicitly inferring network resources, adapting classical or control-based ABR strategies. We, then, provide an overview of the main works focused on implicitly learning the system state, such as reinforcement learning solutions, showing clearly the benefits but also the shortcomings of these solutions. Finally, we describe immersive communication systems in which the adaptation logic needs not only to infer the network resources but also the user behavior over time.

Machine learning allows us to infer complicated relationships between multiple influential factors (e.g., buffer size, network throughput, size of video chunks, the remaining number of chunks) and the bitrate selection decision. Predicting network traffic at the user level is particularly challenging because the traffic characteristics emerge from a complex interaction of user-level and application protocol behavior. Early predictive models were focused on linear ones, such as the autoregressive moving average (ARMA) model [88] and the autoregressive integrated moving average (ARIMA) model [89], used in [90] to predict traffic patterns. Then, non-linear models have been proposed as the ones based on neural network architectures, providing significant improvements in prediction accuracy with respect to the linear counterpart [91]. ANT [92] considered convolutional neural networks to extract network features and classify the network segments automatically. The classifier informs the ABR strategy at the client side. The good inference of non-linear models has also been discussed in [93], in which authors showed that LSTM neural networks outperform the ARIMA models in cellular traffic classification and prediction.

Key challenges that are still under investigation in ABR and that can benefit from learning tools are: (i) fairness in sharing resources across users [94]; (ii) non-stationarity of the streaming environment [93]; and, (iii) low-latency. The well-known network resource sharing problem (distributed Network Utility Maximization (NUM) problems with the goal of sharing resources to maximize the overall users’ benefit) can be solved in a distributed fashion if resource constraints are known a priori, which is not the case in classical DASH systems. This problem is addressed in [95], where authors design an overlay rate allocation scheme that infers the actual amount of available network resources while coordinating the users’ rate allocation. Similarly, collaborative streaming strategies have been presented in [96] for the case of 360-degree video. Users watching the same content form a “streaming flock” and collaborate to predict users’ interactivity levels. The second challenge is mainly focused on the non-stationarity of the data traffic, which makes it difficult and computationally expensive to train a one-size-fits-all predictor/controller. For this reason, in [97] a meta-learning scheme has been proposed consisting in a set of predictors, each optimized to predict a particular kind of traffic, which provide a prior for the adaptive streaming strategy. In [98,99], the critical aspect of an accurate prediction in low-latency streaming systems is discussed.

Alternatively, optimal strategies [99,100] exploiting control theory to solve the video streaming problem have been developed with online learning strategies that continually learn to adapt to dynamically changing systems [101,102]. These solutions, however, may rely on an accurate prediction of the network model. To take into account the uncertainty of the inference model, Bayesian neural networks are considered not only to learn a point of estimate but also a confidence region reflecting the uncertainty on the estimate [103]. This leads to robust ABR solution (or worst-case ABR decisions), which have been proven to outperform the solutions that target at the average-case performance in terms of the average QoE as a more conservative (i.e., lower) throughput prediction can effectively reduce the risk of rebuffering when high uncertainty exists in the prediction.

Methodologies that implicitly learn the system model are (deep) reinforcement learning (RL) algorithms, which can be used to automate the selection of representation over time accommodating, in which an optimal mapping between the dynamic states and bitrate selections is learned. The benefits of RL-based ABR algorithms have been shown in several works [104,105,106,107] and tested experimentally in Facebook’s production web-based video platform in [108]. Further works have been carried out also in the context of Variable Bitrate (VBR), which allocates more bits to complex scenes and fewer bits to simple scenes to guarantee consistent video quality across different scenes. In [109], authors proposed Vibra, a deep-RL framework in which the state (input to deep-RL) is characterized by multiple parameters such as network throughput, buffer size, playback stage, size of video chunks, the complexity of video scenes, and the remaining chunks to be downloaded. However, such promising techniques suffer from key shortcomings such as: (i) generalization (poor performance of the learnt model to heterogeneous network and user conditions [110]); (ii) interpretability (partially addressed in [111,112]); and (iii) high-dimensionality (partially addressed in [107]). Beyond RL-based algorithms, classification by means of unsupervised learning has been proven to be highly efficient in adaptive streaming as in the case of [113] in which the current network and client state is considered an input that needs to be classified and hence it finds the best ABR strategy as a consequence. Similarly, encrypted traffic classification has been proposed in [114] to identify 360-degree video traffic. This can be useful for fixed and mobile carriers to optimize their network and provide a better Quality of Experience to final users.

**Figure 5:**
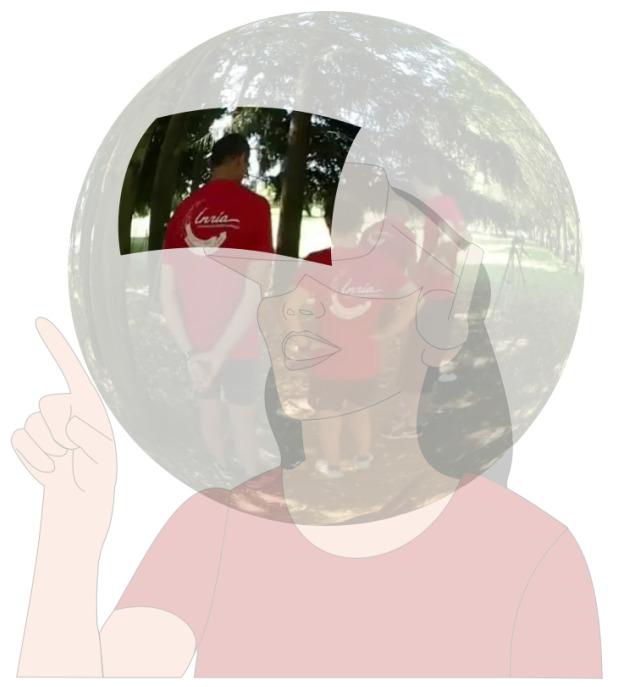
In many applications, including AR/VR/XR and 360-degree video, users are interested in watching a part of a scene (non-shaded area) known as viewport and can freely navigate in the scene enjoying an up to a 6 degree of freedom (DoF) experience.

**Figure 6:**
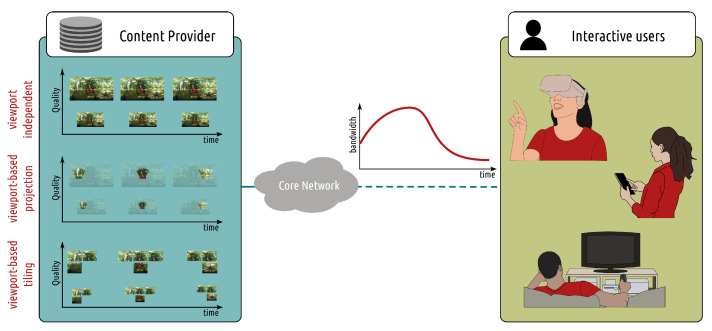
Visualization of different adaptive streaming strategies for interactive systems. In the viewport-independent case, the entire panorama is encoded at multiple quality levels and resolutions and fully sent to final users. The other two approaches are viewport-dependent ones, in which either areas of interest in the panorama are encoded at high quality (viewport-based projection) or the panorama is encoded into multiple tiles and the tiles covering the area to be visualized will be downloaded at higher quality (viewport-based tiling).

Machine learning plays an even more crucial role in the case of interactive communications, in which the dynamic behavior is related not only to network fluctuations but also to users’ movements. Specifically, interactive users watch only a portion of the entire video content (viewport) as shown in Figure 5 and this displayed viewport is driven by users’ movements (such as head’s direction in the case of head-mounted device). To ensure a smooth interaction and a full sense of presence, the video content needs to follow the users’ movements with ultra-low delay. Figure 6 depicts how the generated content might be tailored to interactive users behavior and connectivity. This can be achieved by streaming to each interactive user the full content, from which only a portion will be then rendered. This ensures a low interaction delay at the price of a highly overloaded network. To reduce network utilization, adaptive streaming is combined with tiling to optimize the quality of what is being visualized by the user at a given moment. Tile-based adaptive streaming has been widely investigated in omnidirectional content and more recently also for volumetric content [115,116]. We remind the reader of several excellent review papers on adaptive streaming for immersive communications [10,11,117]. In the following, we mainly focus on the recent advances that made usage of machine learning tools. Initial works have taken into account the user interactivity mainly via probabilistic models [118] or average behavior (e.g., saliency maps) [9] to be used in adaptive streaming strategies. Even if the model of user’s behavior is provided as input to the adaptive streaming strategies, there is still the need to learn the optimal strategy due to the network variations as well as the high-dimensional search space of the optimal solution (not only the bitrates of temporally divided chunks require to be selected, but also the bitrate of every spatially divided tile within a frame should be simultaneously determined based on the dynamics of the viewer’s viewport). In [119], the authors addressed the high-dimensionality of the search space in RL-based adaptive strategies by proposing a sequential reinforcement learning for tile-based 360-degree video streaming. In [120], the combinatorial explosion is rather tackled by proposing a scalable tiling method, in which bit rates are allocated to tiles based on a probabilistic model of the users’ displaying direction, and the optimal adaptation strategy is learned from the RL agent. These works have demonstrated impressive gains in the user’s QoE. Still, most of them learn optimal strategies given that: (i) average users’ behavior is provided (for example, the saliency map) and, (ii) the rest of the pipeline is not tailored to the users as well. This drastically limits the potentiality of the end-to-end pipeline, as shown in [121] and commented in [11]. In the specific case of immersive communications, the interactive user plays a key role and the entire coding-streaming-rendering pipeline should be tailored to each (set of) users, leading to a *user-centric* optimization. The key intuition is that client, server and CDN should be jointly adjusted given the users’ interactivity. In Section 3.5, we better motivate this need showing that users have completely different ways of exploring immersive content, and in Section 4, we further comment on the works that have shown the gain of *user-centric* systems, highlighting what open challenges are still to be solved.

#### 3.3.2. Other Video Streaming Systems

DASH-like video streaming systems permit users to control the adaptation of the video quality based on the encountered channel conditions. However, they face difficulties when are used in a wireless environment where multiple users may have similar demands, as they are designed for point-to-point communication. In wireless environments, it is possible to accommodate multiple users’ requests by a single broadcast or multicast transmission. To this aim, users are grouped based on their requests, which allows serving multiple content requests by a single transmission. Such users grouping is not possible for DASH-like systems, as they cannot exploit the correlation among users’ demands. Further, the emergence of interactive videos like 360-degree videos introduces new challenges as the quality adaptation is made on a tile or viewport basis to save bandwidth resources and meet the strict timing constraints. DASH-like systems cannot exploit videos’ and tiles’ or viewport popularity, and hence they treat each tile as an independent video and deliver only those that are within the requested viewport [122,123]. This approach may be efficient for a single user, but it is suboptimal when multiple users request the same video. Further, DASH-like video streaming systems face difficulties for live 360-degree video streaming, as the video should be prepared in multiple qualities employing possibly cloud farms and then distributed to distribution servers using a CDN infrastructure. This content preparation and delivery may introduce prohibitive delays, as the playback delays are very short for live streaming of 360-degree video. An alternative to DASH-like video streaming is the use of scalable video coding (SVC) [124,125,126]. In SVC-based systems, the video is encoded multiple quality layers (a base layer and possibly multiple enhancement layers). The base layer provides a video reconstruction in basic quality and the enhancement layers progressively improve the video quality. Through SVC coding, the tiles of the 360-degree video that are within a viewport can be reconstructed in high quality, while the rest can be delivered in the base quality.

From the discussion above, it is clear that DASH-like video streaming systems perform suboptimally in wireless environments, as they cannot exploit possible correlations of users’ video content requests. Further, these systems cannot take advantage of broadcasting or multicasting opportunities. This calls for alternative video streaming systems that forecast users’ requests, exploit correlation among video consumption patterns of the users and possible association of users with an access point or base station. As forecasting is central in these systems, machine learning methods have been proposed for both 360-degree video [127,128,129,130,131] and regular video [132,133,134].

The delivery of 360-degree videos captured by unmanned aerial vehicles (UAVs) to a massive number of VR users is studied in [127]. The overlap of the captured videos is exploited to dynamically adjust the access points grouping and decide the communicated video tiles so that VR users’ quality of experience is optimized. As access points only have access to local information, the problem is cast as a Partially Observable MDP (POMDP) and is solved by a deep reinforcement learning algorithm. Due to the fact the complexity of this problem can be extreme, the problem is re-expressed as a network distributed POMDP and multi-agent deep reinforcement learning is proposed to find access points group and tiles transmission schedule. Delivery of 360-degree video to a group of users is also examined in [128] where the optimal download rate per user and the quality of each tile are found by actor-critic reinforcement learning. The presented scheme ensures fairness among users. The impact of various Quality of Service (QoS) parameters on users’ QoE when multiple users request to receive their data through an access point is studied in [129]. The queue priority of the data and the requests are optimized using reinforcement learning. In a virtual reality theater scenario, viewport prediction for 360-degree video is performed using recurrent neural networks and, in particular, gated recurrent units. For proactive 360-degree video streaming, contextual multi-armed bandits are used to determine the optimal computing and communication strategy in [131].

For traditional video streaming, motivated by the fact that higher bitrate is not always associated with higher video quality, in [132] performing a prediction of the quality of the next frames through deep learning to improve video quality and decrease the experienced latency is proposed. A CNN is used to extract image features and an RNN to capture temporal features. A sender-driven video streaming system is employed in [133] for improved inference. A deep neural network is used to extract the areas of interest from a low-quality video. Then, for those areas, additional data are streamed to increase the quality of the streamed video and enhance inference. The available bandwidth and the maximum affordable quality are extracted by a Kalman filter. Reinforcement learning is used in [134] to estimate the end-to-end delay in a multi-hop network so that the best hop node is selected for forwarding the video data.

### 3.4. Caching

The proliferation of devices capable of displaying high-quality videos fueled an unprecedented change in the way users consume data, with portable devices being used for displaying videos. Nowadays, the time users spend on video distribution platforms such as YouTube Live, Facebook Live, and so forth, has surpassed by far the time spent watching TV. This puts tremendous pressure on the communication infrastructure, and as a response, technologies such as mobile edge caching and computing have become an instrumental part of the complex multimedia delivery ecosystems. 5G and beyond networks are comprised of multiple base stations (pico-, femto-, small-, macro-cell) with overlapping coverage areas. As users’ demands on video content are not uniform, but a few popular videos attract most of the users’ interest, caching of videos at the base station became an attractive option to reduce the cost of retrieving the videos. Through caching, the number of content requests directly served by distant content servers is greatly reduced. This is very important as the backhaul links the base stations use to access the core network are expensive and have limited capacity. Further, the core network can be easily overwhelmed by the bulk of requests, which may lead to a collapse of the communication infrastructure. This, in turn, can result in high delivery delays that will be devastating for users’ quality of experience.

**Figure 7:**
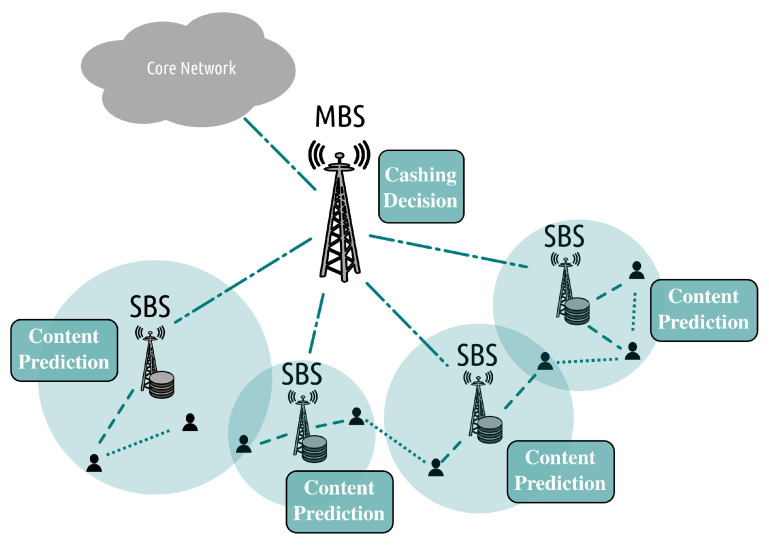
Intelligent caching network. Machine learning is used for content prediction and deciding which content to cache in each SBS and from where to deliver it to the users. Decisions can be centralized at the MBS or distributed at the SBS or follow federated learning concepts.

Content placement, that is, deciding what video content to cache at the base stations, is closely associated with content delivery, that is, from where to deliver the video content to the users. This happens as base stations’ coverage areas overlap, while multiple base stations may possess the same content. These problems can be either studied independently or jointly, but treating these problems separately is suboptimal. Cache placement is often solved using heuristic algorithms such as the Least Recently Used (LRU), Least Frequently Used (LFU), and First-in First-out (FIFO). Alternatively, the caching and delivery problem can be cast as a knapsack problem and then be solved using approximation algorithms [135]. These approximation algorithms cannot solve large-scale problems involving a large number of videos and base stations due to their inherent complexity. Further, the majority of these caching schemes have not been optimized for video data (which constitutes the majority of the communicated content) as they are oblivious to the communicated content and the timing constraints. In order to cope with these limitations, machine learning has been proposed as an alternative way to solve these decision-making problems [12,13]. A high-level representation of an intelligent caching network is depicted in Figure 7, where machine learning methods are used for caching and delivery decision-making and for content requests prediction. There exist multiple surveys discussing the use of machine learning for solving caching and delivery problems [12,13,14]. In this survey, we do not target to cover extensively the caching literature, but we aim at shedding light on recent advances in that field while pointing out the inefficiencies of existing methods toward realizing an end-to-end video coding and communication system. The caching algorithms can be classified into two main categories, namely reactive and proactive caching [135]. In the former category, the content is updated after content requests have been revealed, while in the latter one, the content is updated beforehand. Proactive caching is closely related to prediction problems, while reactive caching is often used to solve content placement.

Different machine learning algorithms have been proposed to solve the content placement and update problem at caches (and in some cases the delivery problem), such as Transfer learning [136,137], deep Q-learning [34,138,139,140], Actor-Critic [141,142], multi-agent multi-armed bandits (MMBAs) [143,144], 3D-CNN [145], LSTM networks [146,147], among other methods. Reinforcement learning algorithms are often preferred, as cache updates can be modelled as a Markov Decision Process [34,138,139,140,141,142,143,144], while deep learning supervised learning methods [145,146,147] are used to capture the trends in the evolution of the video requests. These trends are then used to optimize the content placement and the cache updates. The vast majority of existing machine learning-based caching systems are appropriate only for video downloading [34,138,139,142,148,149,150] as they ignore the tight delivery deadlines of video data. Specifically, they assume that the entire video is cached as a single file, and hence, they ignore timing aspects. The same happens when video files can be partially cached at the base stations by means of channel coding or network coding [135]. In such coded caching schemes, users should download a file with size of at least equal size to the original video file prior to displaying it and hence cannot respect time delivery deadlines. Only a few works in the literature consider timing constraints and can be used for video-on-demand [126] and live streaming [140,141,147].

Most of the existing caching frameworks treat the data as elastic commodities and thus target to optimize the cache hit rate, which is a measure of the reuse of the cached content. Though optimizing cache hit rate can be efficient for file downloading, it is not appropriate for video streaming. This is because it cannot capture critical parameters for video communication such as the content importance and QoE metrics such as video stalling, smoothness, among others, and the relations among the users who consume the content. The latter is considered in [136,137] where information extracted by social networks is used to decide what content should be cached at the base stations. Similarly, contextual information such as users’ sex, age, etc., is exploited to improve the efficiency of caching systems [143]. Although these works improve the performance of traditional caching systems, they remain agnostic on the content. Performance gains can be noticed by clustering the users using machine learning to decide how to optimally serve the content [145] from the base stations and by taking into account QoE metrics [141]. Further performance improvements can be noticed by considering the content importance [126,140,147].

More recently, new types of videos, such as 360-degree videos became popular. The emergence of these video types raises new challenges for caching systems. Specifically, they should take into account how the content is consumed by the users [126]. Caching the entire video requires extreme caching resources, as 360-degree videos typically have very high resolution. Besides, this is not needed as only some parts of the video are popular while the rest are rarely requested. This happens as the users are interested in watching only a part of the video, i.e., a viewport. Therefore, caching algorithms for 360-degree videos should not only consider the video popularity but also take into account the viewport’s popularity or the tile’s popularity. Motivated by this change, the works in [140,147] proposed machine learning algorithms that optimize which tiles to cache and on what quality. Caching systems designed for regular videos [34,138,139,140,141,142,143,144,145] perform poorly for 360-degree video, as they do not take into account the users’ consumption model and the content importance.

Looking forward, methods such as those proposed [140,141,147] can be an integral of future machine learning-based end-to-end video communication systems. However, there are challenges before such methods are adopted. This is due to the fact that the videos will not be compressed by traditional video codecs but by machine learning-enabled video codecs that generate latent representations. This creates new challenges in building algorithms that decide what content should be cached and where.

### 3.5. QoE Assessment

Inferring the human perceptual judgment of media content has been the goal of many research works for many decades as it would enable optimizing multimedia services to be QoE-aware/centric [151,152]. Here, we do not intend to provide an exhaustive literature overview on QoE, while we rather focus on ML advances in the field of QoE.

Quantifying quality of experience requires the definition of one or more metrics that are specific to the context, and equally important, a formal way of computing the values for these metrics. Only after this, we can measure a human’s level of satisfaction from the respective service or application. One major challenge in the field of quality assessment has been the model inference in the case of no-reference quality assessment, in which no information about the originating content is provided. Before the deep-learning era, hand-crafted features such as DCT, steerable pyramids, etc., were carried out to then build a perceptual model. With the advances of deep learning, automated features extraction has been considered, obtaining reliable semantic features from deep architectures (e.g., CNNs) achieving remarkable efficiency at capturing perceptual image artifacts [153,154,155,156]. This however requires a large-sized labeled dataset, which is expensive (and not always feasible) to collect. Transfer learning has been adopted to learn feature extraction models from well-known image datasets (such as Imagenet) and then fine-tune the current model on subjectively tested dataset [157]. As an alternative, the recent ML advances on contrastive learning [158] and self-supervised learning [159] might have opened the gate to self-supervised quality assessment.

Learning which of these extracted features play a key role in the human perceptual quality is a second challenge to be addressed. VMAF [160] proposed this, and it has been extended to 360-degree content. Specifically, video quality metrics are combined to human vision modeling with machine learning: multiple features are fused together in a final metric using a Support Vector Machine (SVM) regressor. The machine-learning model is trained and tested using the opinion scores obtained through subjective experiments. Moreover, convolutional neural networks have also been considered to fuse multi-modal inputs. For example, motivated by the fact that image quality assessment (IQA) can be greatly improved by incorporating human visual system models (e.g., saliency), in [161] an end-to-end multi-task network with a multi-hierarchy feature fusion has been proposed. Specifically, the saliency features (instead of saliency map) are fused with IQA features hierarchically, improving the IQA features progressively over network depth. The fused features are then used to regress objective image quality.

Assessing QoE is even more challenging in the case of video content [162], in which each frame QoE is evaluated and then a temporal pooling method is applied to combine all frames’ quality scores. Another solution is to train an LSTM network able to capture the motion and dynamic variations over time [163]. The problem of QoE assessment in video is amplified in the case of video such as mobile videos, drones, crowd-sourced interactive live streaming, and so forth, with such high-motion content that strongly affects the perception of distortions in the videos. Machine learning can drastically help toward this goal in extracting dynamic features that can learn semantic aspects of the high-motion content relevant for the final user QoE [164].

Despite all the research carried out on ML for QoE, there are still open challenges in assessing human quality in the experienced streaming services. As observed by multiple researchers, QoE goes beyond the pure quality of the played video and hinges on the viewer’s actual viewing experience. Current methods primarily use explicit information such as encoding bitrate, latency and quality variations, stalling, but they do not make use of less explicit yet more complex intra-session (e.g., viewer’s actions during the streaming session, such as stopping, pausing, rewinding, fast-forwarding, seeking, switching to another channel, movie, show, etc.) and inter-session relationships (e.g., variation in the inter-session times of the viewer and their hourly/daily/weekly viewing times). Cross-correlating actions and change of habits among different viewers is also effective in gaining significant insight into human satisfaction of the streaming service, which is still a measure of QoE. Machine learning can indeed help toward this goal. Another important challenge to mention is the bias that comes in training ML model (to extract and fuse features) via subjective tests. As pointed out in recent and very interesting panels [165] and works [166], the impact of bias and diversity in the training dataset is a key challenge in machine learning and it can therefore have an impact on QoE—leading to a model that fits mostly part of the population but ignores minorities.

New challenges have emerged when new multimedia formats have been introduced, such as spherical and volumetric contents. The entire compression and streaming chain has been revisited for these formats. It is important to understand the impact of compression and streaming on the quality of spherical and volumetric content [167]. In [168], a spatio-temporal modeling approach is proposed to evaluate the quality of omnidirectional video, while in [169,170,171] quality assessment of the compressed volumetric content is studied for two popular representations (meshes and point clouds). The results show that meshes provide the best quality at high bitrates, while point clouds perform better for low bitrate cases. In [172], the perceived quality impact of the available bandwidth, rate adaptation algorithm, viewport prediction strategy and user’s motion within the scene is investigated. Beyond the coding impact, the effect of streaming on QoE has also been investigated. In [173], the effect of packet losses on QoE is investigated for point cloud streaming. Still, in the case of point cloud streaming, in [172] authors investigated which of the streaming aspects has more impact on the user’s QoE, and to what extent subjective and objective assessments are aligned. These key works have enabled the understanding of the impact of different features and format geometry on QoE. Since also in 360-degree streaming the QoE is dependent on multiple features [174], ML can help toward automating these steps. In [10], advances toward this direction have been summarized.

### 3.6. Consumption

An interesting question arising is how the streaming pipeline should be optimized depending on how we consume content. Specifically, lately, we have witnessed two main revolutions in terms of consumption: (i) the final user consuming the content is no more limited to be a human but can be a machine performing a task; (ii) the final user (when human) is able to actively interact with the content, creating a completely new type of service and, hence, streaming pipeline optimization. The first direction is in its infancy and we further comment on this in the following section. Conversely, several studies have already focused on optimizing the streaming pipeline in the case of interactive users, as already mentioned in the previous sections. These efforts however have relied on a key piece of information, which is the behavior of the users during interactive sessions. We refer the reader to many interesting review papers on user’s behavior [10,11]. Here, our focus is mainly on highlighting the key machine learning tools that have been used to improve saliency prediction.

The first efforts at understanding user behavior were focused on inferring the *saliency map* for 360-degree content and using this information in the streaming pipeline [175]. The saliency map is a very well-known metric that maps content features into user attention; it estimates the eye fixation for a given panorama. One of the first end-to-end trained neural networks to estimate saliency was proposed in [176], in which a CNN was trained to learn and extract key features that are relevant to identify salient areas. These models, however, were trained mainly with only a single saliency metric. To make the model more robust to metric deviations, SalGAN [177] has been proposed, which employs a model trained with an adversarial loss function. These approaches have been extended for 360-degree content. For example, two CNNs have been considered in [178], where a first SalNet network predicts saliency maps from viewport plane images projected from 360-degree images, and a subsequent network refines the saliency maps by taking into account the corresponding longitude and latitude of each viewport image. The SalGAN model was extended to SalGAN360 [179] by predicting global and local saliency maps estimated from multiple cube face images projected from an equirectangular image. Instead of adversarial loss, multiple losses have been used during training in [180] and a neural network is used to extrapolate a good combination of these metrics. Looking also at the temporal aspect of the content, LSTM networks have been proposed [181], in which the spherical content is first rendered on the six faces of a cube and, then, concatenated in convolutional LSTM layers. Interestingly, ref. [182] studied the effect of viewport orientation on the saliency map, showing that the user attention is affected by the content and the location of the viewport. Authors proposed a viewport-dependent saliency prediction model consisting of multi-task deep neural networks. Each task is the saliency prediction of a given viewport, given the viewport location, and it contains a CNN and a convolutional LSTM for exploring both spatial and temporal features at specific viewport locations. The outputs of the multiple networks (one per task) are integrated together for saliency prediction at any viewport location. Recent studies have also used machine learning for predicting saliency in the case of multimodal inputs such as both video and audio features [183]. All the above works study saliency on spherical data by projecting the data into a planar (2D) domain, introducing dependency of the learned models from the projection as well as deformation due to the projection. Instead of working on the projected content, ref. [184] proposed a spherical convolutional neural network for saliency estimation. Similarly, ref. [185] proposed a graph convolutional network to extract salient features directly from the spherical content. Beyond 360-degree content, volumetric images and video have been under deep investigation from the users’ behavior perspective. Saliency for point clouds has been studied in [186,187,188], and machine learning tools can play a key role in this very recent research direction.

Instead of looking at saliency, we now review works that use machine learning to study users interactivity via *field of view trajectory*. In this direction, machine learning has helped toward two main directions: (i) to group similar users by using unsupervised tools such as clustering; (ii) to predict future trajectories of users interactivity. Looking at the first direction, detecting viewers who are navigating in a similar way allows the quantitative assessment of user interactivity, and this can improve the accuracy and robustness of predictive algorithm but also allow the personalization of the streaming pipeline. Last, this quantitative assessment can play a key role also in healthcare applications, in which patients can be assessed based on their eye movement when consuming media content [189]. Looking at the user navigation as independent trajectories (e.g., tracking the center of the viewport displayed over time), users have been clustered via spectral clustering in [190,191]. A clique-based clustering algorithm is presented in [192], where a novel spherical clustering tool specific for omnidirectional video (ODV) users has been proposed. With the idea of building meaningful clusters, the authors proposed a graph-based clustering algorithm that first builds a graph in which two users are connected with a positive edge only when looking at the same viewport. Then, a clique-based algorithm is considered to identify clusters of users that are all connected with each other. This ensures that all users within the cluster experience almost the same viewport.

Beyond users similarity, several works focus on predicting user behaviors via deep learning strategies that learn the non-linear relationship between the future and past trajectories. The prediction is usually adopted for viewport-adaptive adaptation logic, but it can also be used to optimize the rate-allocation at the server side as in [122,193,194]. CNN [194], 3D CNN, LSTM [123], Transformers [195], and deepRL [196] frameworks have been considered to predict users’ viewport and improve the adaptation logic, as highlighted in the recent overview [chapter]. The work in [194] develops a CNN based viewport prediction model to capture the non-linear relationship between the future and past trajectories. 3D-CNN is adopted in [193] to extract spatio-temporal features of videos and predict the viewport. LSTM networks are instead used to predict future viewport based on historical traces [197]. Beyond looking at the deep learning network used for the prediction, existing works can be categorized into multi- and single-model inputs. The latter ones are limited to historical data trajectory for the viewport prediction. Conversely, multi-modal input prediction can be considered in the case in which the input of the neural network is not only the user head position but also video content features, motion information, object tracking [198] as well as attention models [199]. Interestingly, recent works [195,200,201] have shown that multi-modal learning systems, which are usually composed of neural networks and, therefore, require heavy computation and large datasets, are not necessarily achieving the best performance. Transformers have been used in [195] in which the deep learning framework is trained with the past viewport scanpath only, and yet it achieves a state-of-the-art performance.

## 4. Towards an End-to-End Learning-Based Transmission System

The learning-based methods reviewed in Section 3 clearly lead to unprecedented performance gains in data transmission. However, these improvements do have a price. On top of overall complexity growth, most of the algorithms presented above come with a paradigm shift and, therefore, with potential compatibility issues with some other modules of the transmission chain. Such a revolution thus has to be considered on the entire communication pipeline at the same time. In this section, we first argue why the advent of learning-based solutions imposes a global revolution of the full chain and we review the first efforts in the literature in that direction. Second, we expose how learning-based approaches maybe a cornerstone of this revolution.

### 4.1. ML as Cause of the Revolution

*Latent vector description of the images/video*: as explained in Section 3.1, learning-based image/video coders produce a bitstream that is the direct entropy coding of a single latent vector describing the whole image (see Figure 3). It is quite obvious that such a bitstream is not compatible with the current standards. Even worst, the decoding architecture is, each time, specific (in terms of structure and weights), and a standardization of the bitstream is therefore hardly conceivable. Therefore, the usability of such learning-based coders is put in question. Indeed, it is obviously impossible in practice to build decoding hardware in which the decoding process is not unified. For example, the decoder needs to know the layer architecture along with the learned weights, which implies to transmit them at some point. Solutions have been investigated to compress the weights of neural networks architecture, as reviewed in [202]. The principles of such methods are for example a sparsification of the network or a weight quantization. Even though this can indeed decrease the model cost, this might be insufficient when dealing with the huge architecture employed for data compression. Future work may consider the cost of model transmission in the network loss definition.

Another important consequence of the latent-based description is that the standard hierarchical description of the bitstream in the Network Abstraction Layer (NAL) is not defined (and not straightforwardly definable), which makes the streaming of such a binary description not compatible with the standard methods (e.g., DASH). Even though some learning-based coders let the core of their architecture relying on standard codecs (such as in [203]), future compression methods may consider the transmission aspects during the coding strategy in more depth.

**Figure 8:**
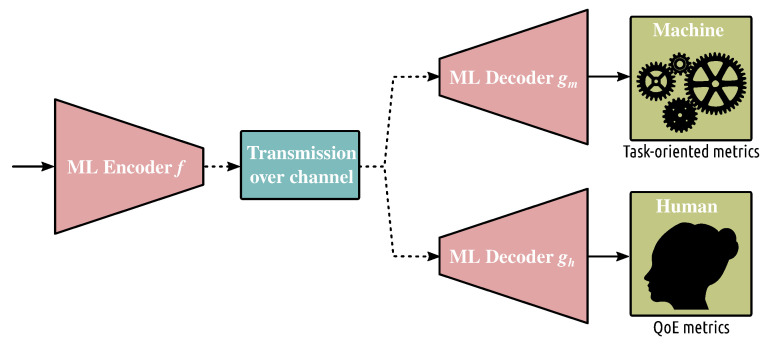
Compression aim changes since the decoded images or videos can be used to perform automatized tasks (e.g., identify dangerous situations in vehicular networks, recognize persons, etc.) apart from being watched by humans. This necessitates the consideration of different metrics for defining the loss functions of the neural network architectures.

*Coding for machines*: the tremendous amount of transmitted images/videos nowadays are simply not meant to be watched by humans, but to be processed by powerful learning-based algorithms for classification, segmentation, post-processing, and so forth (as illustrated in Figure 8). However, machines and humans think and elaborate the perceived environment differently. A random noise barely perceived by human eyes can lead to mis-classification from a deep network [204]. At the same time, machines can correctly perform binary classification on filtered images, in which the subject is barely visible to the human eye [205]. Even though task-aware compression has been investigated for a long time, we can clearly see a real need for compression aim redefinition due to the explosion of learning-based processing algorithms. In [2], new use cases are investigated. More concretely, three decoders are considered: (i) for standard visualization aim (the loss is PSNR, SSIM or any subjective assessment metric), (ii) for image processing task (enhancement, editing), or (iii) for computer vision task (segmentation, recognition, classification, etc.). Most of the time, these processing tasks are deep learning-based, and the corresponding losses totally differ from the standard ones. In the same spirit, the MPEG activity on Video Coding for Machines (VCM) has been established to build new coding frameworks that enable lower bitrates without lowering the performance of machine analysis, e.g., autonomous vehicle driving [206]. Pioneering works have investigated the effect of video codec parameters on task accuracy, preserving however the MPEG standard codecs [207]. For such problems, the difficulty naturally comes when several processing tasks are considered, or even worse, when the processing task is unknown at the encoding stage. Some works have been proposed for image compression [208,209] or video streaming protocol redefinition [133]. In [210,211], features-based compression algorithms aim at maximizing quality perceived both by humans and machines.

### 4.2. ML as Solution to the Revolution

*End to end video coding and communication:* The benefits of redesigning the traditional image coding and transmission pipeline using a deep neural-based architecture have been first demonstrated in [212] where deep JSCC was presented. Deep JSCC is a joint source and channel coding scheme designed for wireless image transmission. In this scheme, similar to the deep image compression schemes discussed in Section 3.1, the DCT or DWT, the quantization, and the entropy coding are replaced by a deep neural network architecture, but in addition, it implicitly considers channel coding and modulation together with the image coder to introduce redundancy to the transmitted bitstream so that it can combat channel impairments. The encoder and the decoder are parametrized into two convolutional neural networks, whereas the noisy communication channel is modeled by a non-trainable layer in the middle of the architecture. This architecture is inspired by the recent success of both deep image compression schemes [209] and deep end-to-end communication systems [76], which are often based on autoencoders. Deep JSCC achieves superior performance to traditional image communication systems, particularly in the low signal-to-noise ratio regime and even more interestingly shows improved resilience to channel mismatch. Further, deep JSCC does not suffer from the “cliff effect”, where rapid degradation of the signal quality is noticed when channel conditions deteriorate beyond a level. It also continues to enhance the image quality when the channel conditions improve. This is in contrast to traditional wireless image communication systems, where the systems are commonly designed to work targeting a single image quality or multiple qualities and cannot take advantage of when channel conditions are better than anticipated.

This superior performance of deep wireless image communication systems renders deep video coding and communication architectures promising candidates for future video communication systems. However, the application of such architectures to video communication is not straightforward. This is because video coders aim to remove not only the spatial redundancy but also the temporal redundancy for even greater compression gains, which introduces more dependencies. There are further challenges as the video coding pipeline is more complex than the image coding one. Therefore, mapping the entire pipeline to a single deep neural network architecture may not be trivial or efficient. Challenges also arise because of the delay-sensitive nature of video communication, which should be considered to avoid excessive delays. Even if mapping the entire video coding and transmission pipeline to a single deep neural network architecture is not possible, and only parts of it can be replaced, these should be holistically considered and not in a fragmented way. Finally, supporting progressivity or embedded encoding in variable bitrates are well-desired features of future video communication systems. However, while the benefits of using deep learning architectures over traditional methods to achieve progressivity [213] or supporting variable bitrates [214,215] have been demonstrated for image coding, their application to video is not straightforward due to the inherent complexity of the video coding pipeline.

*Users-centric systems*: As observed in Section 3, immersive communications created new challenges around the interactivity of the users with the content. A promising solution is to develop personalized VR systems able to properly scale with the number of consumers, ensuring the integration of VR systems in future real-world applications (user-centric systems). This is possible by developing an efficient tool for navigation patterns analysis in the spherical/volumetric domain and leveraging that to predict users’ behavior and build the entire coding-delivery-rendering pipeline as a user-and geometry-centric system. Several learning mechanisms have been adopted to predict/understand users’ viewport (as discussed in Section 3.5), here we mainly focus on the adoption of these inferred models to the different steps of the pipeline. Saliency prediction models have been used in various multimedia compression applications such as rate allocation [216], adaptive streaming [217], and quality assessment [218] too. Looking more at viewports’ prediction and its impact on the pipeline, the authors of [123] combined a CNN-based viewport prediction with a rate control mechanism that assigns rates to different tiles in the 360-degree frame such that the video quality of experience is optimized subject to a given network capacity. In [219], users’ viewport prediction is instead used to predictive rendering and encoding at the edge for mobile communications. Other works mainly focus on the prediction error and/or uncertainty to tailor the rate-allocation strategy [122,194]. Another line of works was proposed to integrate users’ prediction in Deep Reinforcement Learning (DRL)-based adaptation logic [120,193,220,221]. These works have demonstrated promising potentials in improving streaming experiences [116]. However, one of the main limitations of current viewport-adaptive streaming strategies is that they suffer from aggressive prefetching of tiles, hence do not respond well to the highly dynamic and interactive nature of users viewing 360-degree video content. There is actually a major dilemma for current 360-degree video streaming methods to achieve high prefetching accuracy versus playback stability. A key insight is that there is no a “one-size-fits-all” solution to strike this tradeoff as the optimal balance between prefetching and playback stability is highly dependent on the users’ behaviors. As observed in [222], users tend to interact in different ways despite the displayed content: some users are more randomly interacting with the content, others tend to be more still, while few others have the tendency to follow the main focus of attention in the media content. Following this line of thought, ref. [121] proposed an adaptation logic that is tailored to the QoE preferences of the users. Specifically, the authors propose a preference-aware DRL algorithm to incentivize the agent to learn preference-dependent ABR decisions efficiently.

Despite the gains achieved so far, most of the existing works exploit the users’ inferred model in one step of the pipeline, limiting the potentiality of user-centric systems. *A key open challenge is to tailor the entire end-to-end pipeline to users model*. For example, in the case of tile-based adaptive streaming in 360-degree video, user-centric adaptation logic should be optimized jointly with the tiling design, similarly for the CDN delivery strategy. Moreover, we should re-think the whole coding and streaming pipeline taking into account the interplay between motion vector within a scene (content-centric) and the users’ interactivity vector (user-centric).

## 5. Conclusions

The introduction of machine learning into several parts of the multimedia communication pipeline has shown great potential and has helped to achieve tremendous performance gains compared to conventional non-machine learning-based multimedia communication systems. As a result, machine learning-based components have already replaced parts of the conventional multimedia communication ecosystem. However, besides the performance gains, machine learning faces difficulties when it is used in other parts of the ecosystem due to compatibility issues with legacy multimedia communication systems. For example, the generated image/video bitstreams by machine learning-based codecs cannot be decoded by conventional codecs. Another example is related to the transport of the bitstreams into packets, as typically latent vectors are large in size. Hence, these issues should be resolved before machine learning becomes an essential part of the entire multimedia communication ecosystem. Initial efforts to build machine learning-enabled end-to-end image communication systems showed great performance gains. However, so far, the introduction of machine learning to the entire video communication pipeline is still in its infancy, and efforts have only targeted parts of the pipeline in a fragmented way. This is a necessity as even 5G networks cannot support bandwidth-killing applications based on videos such as XR/AR/VR, which involve the communication of huge amounts of visual data and are characterized by strict delivery deadlines. Moreover, the realization that the vast majority of the communicated video is not intended to be watched by humans calls for jointly studying existing machine learning-based video coding approaches in order to build approaches appropriate for both machine-oriented and human-centric systems. Towards this goal, machine learning will be instrumental in finding the optimal tradeoff between content-centric and human-centric objectives. 

## Data Availability

Not applicable.
